# Photoelectron Circular Dichroism in the Photodetachment of Deprotonated 1-Phenylethanol

**DOI:** 10.1021/acs.jpclett.4c03636

**Published:** 2025-02-03

**Authors:** Viktoria K. Brandt, André Fielicke, Gerard Meijer, Mallory L. Green

**Affiliations:** Fritz Haber Institute of the Max Planck Society, Faradayweg 4-6, 14195 Berlin, Germany

## Abstract

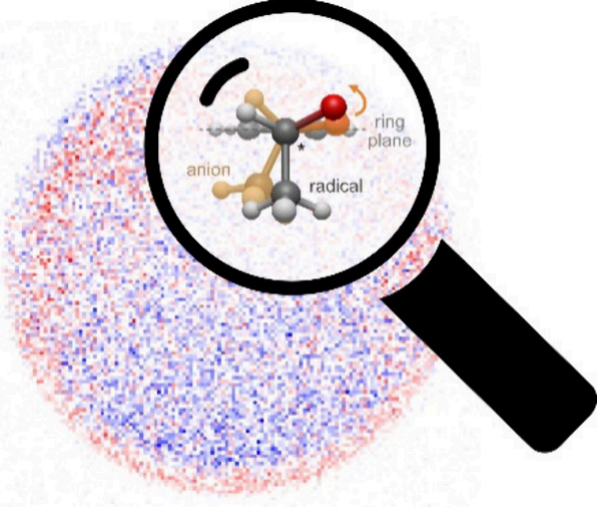

Photoelectron circular dichroism (PECD) is a chiroptical effect that manifests in the angle-dependent photoemission of an electron upon irradiation of a chiral molecule by circularly polarized light. Studies of this chiroptical effect can aid in our fundamental understanding of electron dynamics, as this effect is acutely sensitive to the probed molecular state and electron emission conditions. Photodetachment of anions is a photoemission regime that has historically been understudied in conjunction with PECD. Through comparisons to electronic structure calculations, the photoelectron spectrum of deprotonated 1-phenylethanol is assigned to a single electronic transition of a single conformer. This allows for the investigation of the dependence of PECD on the electron kinetic energy and vibrational state of the molecule. PECD in the photodetachment of the deprotonated 1-phenylethanol anion is measured at different photon energies from 3.59 to 2.38 eV, showing a change in the resulting PECD value for a given transition with differing electron kinetic energies.

Chiroptical spectroscopies in the gas phase are of growing interest in fields of analytical and physical sciences. In particular, photoelectron circular dichroism (PECD) spectroscopy is of interest due to the possibility that it can be used to investigate dilute, multicomponent chiral samples through coupling with mass spectrometry, as well as its potential for unraveling questions in electron dynamics.^1−7^ In a measurement of PECD, photoelectrons are detached from randomly oriented chiral molecules in the gas phase using circularly polarized light. The chiroptical effect measured in PECD is the forward/backward asymmetry of the emission of these photoelectrons, which inverts with a change in enantiomer or the handedness of circularly polarized light.^8−10^ PECD is acutely sensitive to the state and geometry of the probed molecule, as well as the conditions of photoemission, such as the departing electron kinetic energy.^11−22^ This is due to the scattering of the electron across the molecular potential as it is emitted. The sensitivity of this effect to the molecular potential exceeds that of both the photoionization/detachment cross section and the anisotropy parameter (β) and enables the investigation of even vibrational dynamics, despite the Franck–Condon principle.^23−27^ The ability to capture vibrational dynamics provides an avenue for exploring mechanisms of induced chirality and enantioselective reactivity.^5,7,28,29^

As the field of PECD measurements continues to grow, so does the need for complementary theory to understand and make accurate predictions of this effect. However, due to the numerous influential forces in the short- and long-range interactions between the molecule and the electron, modeling of this effect can be more complicated than the theory supporting other chiroptical effects. This difficulty results in a trade-off between accuracy and molecule size. B-Spline and CMS-Xα scattering methods are both popular methods used in the prediction of PECD for real molecules.^10,11,30,31^ However, they are known to suffer from inconsistencies with near-threshold electrons, where the scattering is the most complicated and electron correlation effects might become more important. Also, these theoretical methods account for only the electronic transition that occurs in the photoemission and are blind to the vibrational change that can occur, to which PECD has been experimentally shown to be sensitive. On the contrary, other theoretical methods can provide a fundamental understanding of the effect but are too computationally expensive to apply to real molecules.^32,33^ In general, experiments dedicated to disentangling the forces that govern the universal dynamics of PECD would serve to further close the gap between the experiment and theory.

Utilizing slow electron velocity map imaging, near-threshold electron detection can be carried out to obtain vibrational energy resolution and accumulation of the full angular distribution of the photoelectrons.^34^ Furthermore, studying the PECD effect in the photodetachment of anions simplifies the electron–molecule interaction forces as the long-range Coulombic interaction is missing. Therefore, only short-range forces require consideration. This agrees also with recent findings based on subcycle resolved strong field ionization of chiral molecules where it was concluded that PECD is caused by the chiral initial state rather than being due to the electron propagating in a chiral ionic potential after tunneling.^35^

There are only a few cases of PECD being observed in anion photodetachment.^36−38^ A publicly available document on the outcome of a grant proposal has reported a PECD for photodetachment of several deprotonated chiral aliphatic alcohols.^39^ Recent theoretical investigation has also supported the case for chiral anions to carry a sizable PECD.^32^ Also, our recent work on the photodetachment of deprotonated 1-indanol demonstrated that the PECD observed in photodetachment of anions can be of similar magnitude compared to photoionization of its parent molecule.^36^ The measured PECD was averaged across multiple overlapping electron detachment channels represented by energetically similar tautomers, conformers, and electronic states, thus limiting our ability to investigate individual contributors to the PECD effect. This work provides a well-resolved study of a prototypical deprotonated chiral molecule, 1-phenylethanol (PhEtO^–^), with a simple electronic structure, where PECD measurements taken at multiple photon energies can provide insight into the effects on PECD under explicitly short-range forces.

Anions within our instrument are formed through deprotonation. Therefore, enthalpies of deprotonation of PhEtOH were calculated for several tautomeric anions that are obtained by deprotonation of the hydroxyl group, the methyl group, and the chiral carbon. Formation of an anion through deprotonation of the methyl group was calculated to be endothermic by 0.105 eV (10.18 kJ/mol) and is not expected to contribute to the photoelectron spectrum. Deprotonations at the hydroxyl group and the chiral carbon are exothermic by −0.958 and −0.398 eV, respectively. These two tautomers are predicted to be well separated in electron detachment energy (eDE), with the vertical eDE of the carbon-deprotonated tautomer expected to be 0.781 eV and that of the oxygen-deprotonated tautomer to be much higher (2.269 eV), enabling a separate analysis of these two species. In Figure 1, the photoelectron spectrum taken at a photon energy (*h*ν) of 3.49 eV shows a strong, broad feature for an eDE of >2.0 eV that corresponds to the O-deprotonated anion and a much weaker feature above 0.5 eV, corresponding to the C-deprotonated anion. The low relative intensity of the latter band is supported by a Franck–Condon simulation of the photodetachment of this tautomer, which shows a small Franck–Condon overlap for this detachment channel. The minimal presence of the detachment of the C-deprotonated tautomer, in conjunction with this tautomer anion being achiral, allows us to exclude it from our PECD analysis. Therefore, the deprotonated phenylethanol anion (PhEtO^–^) refers to the O-deprotonated tautomer, herein.

**Figure 1:**
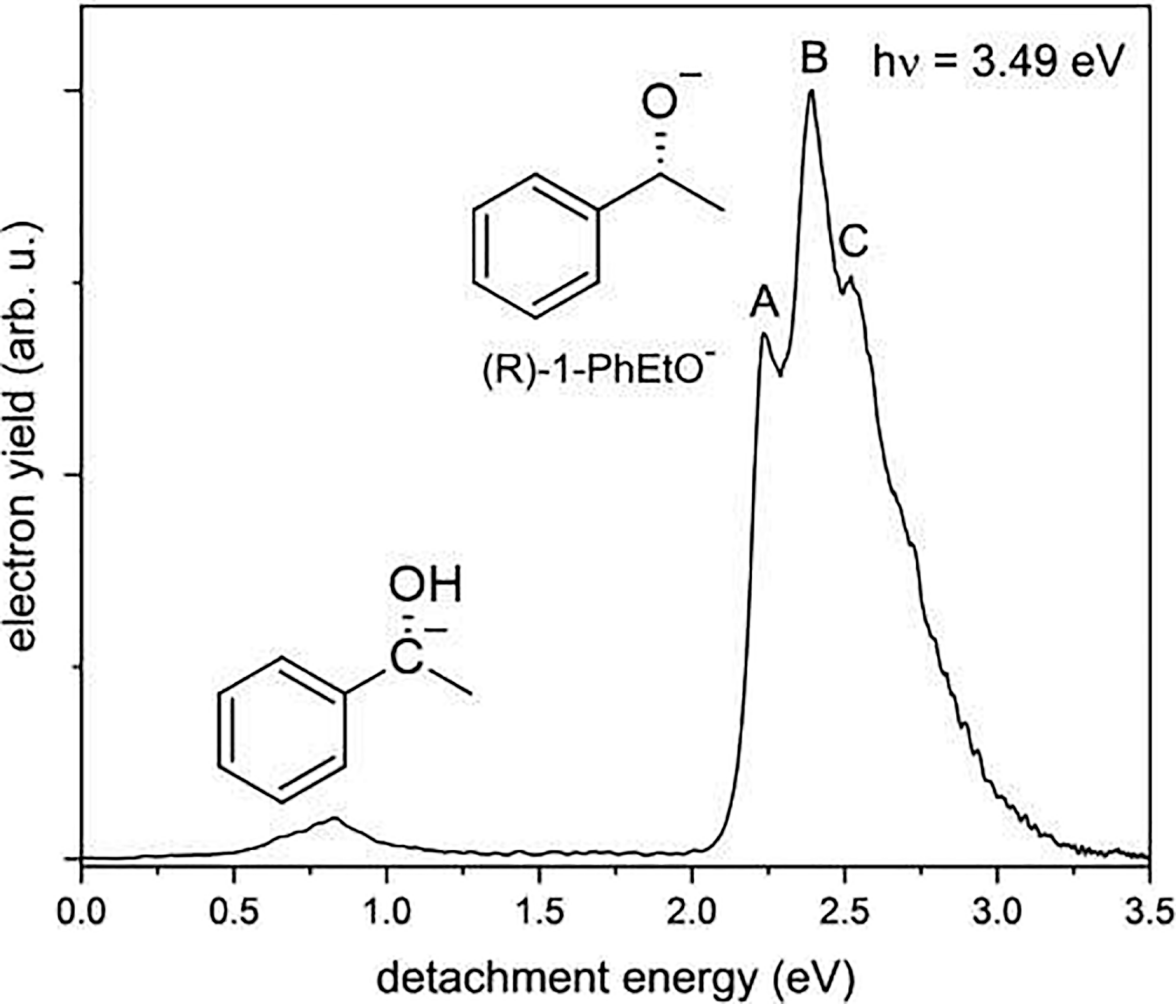
Photoelectron spectrum of the deprotonated (*R*)-1-phenylethanol anion, acquired at a photon energy (*h*ν) of 3.49 eV.

An initial guess of the conformers of PhEtO^–^ was based on the three known conformers of 1-phenylethanol.^40^ Deprotonation at the hydroxyl group results in three possible conformations, resulting from the rotation about the C–phenyl bond (as shown in Figure S2). Optimizations with initial geometries of either the C–O^–^ bond, the C–methyl bond, or the C–H bond in the plane of the ring all yield the conformation in which the oxygen is approximately in the plane, indicating there is a single conformer for the anion. The presence of this conformer can be understood through stabilization stemming from an interaction between the phenyl ring and CO^–^ (see below).

The optimized geometry of PhEtO^–^ was used to generate the Franck–Condon simulated spectrum, at a simulation temperature similar to that of the experimental conditions, shown in Figure 2. The simulated spectrum of the photodetachment from the ground electronic state of PhEtO^–^ is in very good agreement with the photoelectron spectrum taken at a photon energy (*h*ν) of 2.76 eV. The simulation is based on harmonic frequencies. One might expect deviations due to anharmonic behavior arising from low-frequency modes. Nonetheless, the three prominent bands in the experimental spectrum (i.e., A–C) have all been reasonably reproduced. The relative intensities of the three bands in the 3.49 eV photoelectron spectrum in Figure 1 are significantly different from the simulation and the other spectra recorded at different photon energies, possibly due to some unidentified above-threshold resonance.^41−43^ In addition, calculation of the excited states of the neutral radical photoproduct provides a vertical detachment energy to the first excited state of 0.254 eV above the fundamental electronic transition or an eDE of 2.523 eV. This excitation is correlated to detachment from the anion’s HOMO–1 orbital, and given its predicted detachment energy, this excitation is unlikely to contribute significantly to the photodetachment spectrum recorded at 2.76 eV.

**Figure 2:**
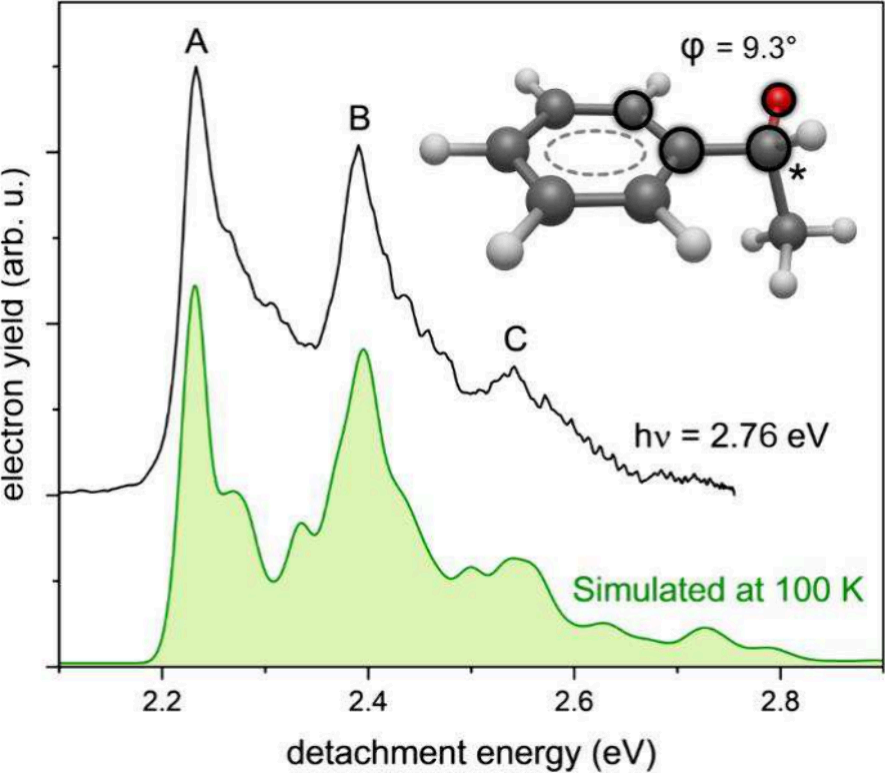
Photoelectron spectrum of the PhEtO^*–*^ anion acquired at a photon energy of 2.76 eV (black) and Franck–Condon simulated spectrum (green). The inset shows the optimized geometry of R-PhEtO^*–*^. The dihedral angle (φ) is defined by the atoms outlined in black. The asterisk marks the chiral center of the molecule.

A closer look at the individual transitions of the simulated spectrum reveals the prevalence of the lowest vibrational mode (ν_1_) within the total vibrational content of the spectral progression. ν_1_ is a low-frequency (40 cm^–1^) mode with torsional motion that corresponds to a rotation around the bond between the chiral carbon atom and the phenyl ring. This twisting motion reflects the transition from the ground state geometry of the anion to the preferred neutral radical geometry, where the oxygen becomes more perpendicular to the ring plane. This observation supports the conclusion of avoidance between the extra electron density at the anionic oxygen and that of the π-cloud. In the neutral radical, the dihedral angle between that of the oxygen and the ring plane is identical to the same dihedral angle for conformer 2 of PhEtOH (i.e., −29°).^40^ Excitation of this vibrational mode upon photodetachment is the primary contributing detachment channel for band A, and bands B and C are comprised of combinations of the ν_1_ mode with higher-frequency vibrational modes. The average number of quanta in the ν_1_ mode is three for the fundamental and combination bands. Therefore, it can be assumed that the presence of the ν_1_ mode contributes to the overall broadening of the entire spectrum.

The additional vibrational modes contributing to the vibrational content of peaks B and C are primarily vibrational modes ν_24_, ν_25_, ν_27_, ν_28_, ν_30_, ν_31_, and ν_33_. These modes largely represent vibrational motions of C–H deformations, and C–C stretches, mostly in the plane of the ring. In addition, modes ν_24_, ν_25_, ν_30_, and ν_33_ show some C–O stretch character. Peak B is composed of combination bands containing one quanta of one of these higher-frequency modes and the ν_1_ contribution. Peak C contains combination bands of two higher-frequency modes with one quanta each, or in the case of ν_30_ two quanta, and the ν_1_ contribution. Tables of vibrational frequencies of the neutral radical and a table of the contributing vibrational modes for each spectral band can be found in the Supporting Information.

Photoelectron spectra and PECD, measured for both enantiomers of PhEtO^–^ at four photon energies, are shown in Figure 3. Measured PECD is shown for the regions of the photoelectron spectra where the electron yield is at least 10% of the maximum electron yield recorded for the spectrum. The confidence intervals of the PECD measurements are the standard error determined through averaging of multiple measurements for the *R* enantiomer at a given photon energy. In the *h*ν = 3.49 eV spectrum, a standard error determination is also provided for the *S* enantiomer, but for all other photon energies, the *S* enantiomer simply serves to confirm the expected sign reversal of the effect. PECD determined at the electron yield maximum of each peak is provided in Table 1.

**Figure 3:**
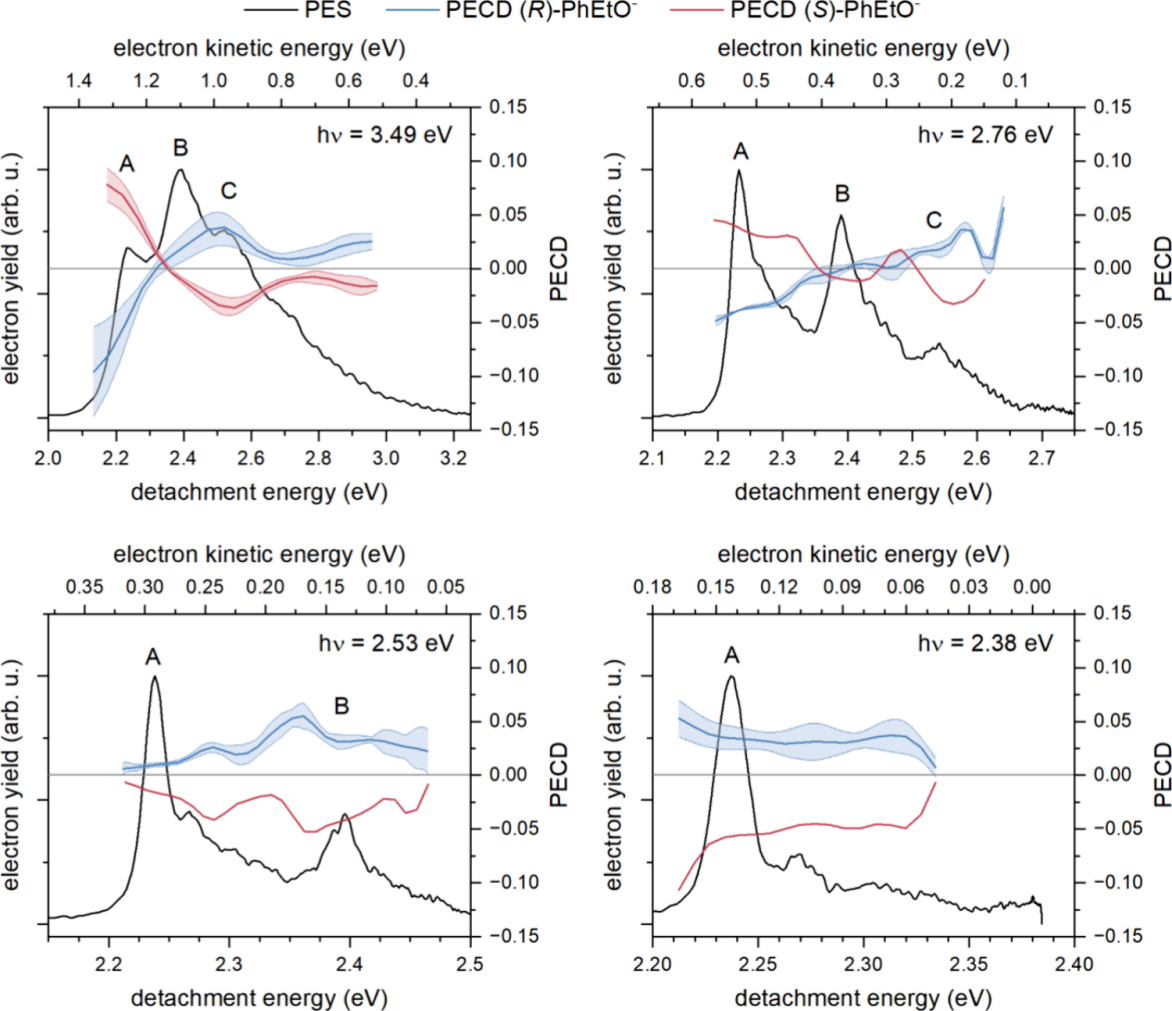
Photoelectron spectra (black) and PECD (blue for *R* and red for *S*) of the PhEtO^*–*^ anion measured at photon energies ranging from 3.49 eV (top left) to 2.38 eV (bottom right). Light red and blue shadings indicate error ranges of the PECD measurement.

**Table 1:** Peak Electron Detachment Energies (eDE) and PECD Measured at the Maximum of Each Peak (A–C) at Four Photon Energies (*h*ν) for the *R* Enantiomer

eDE (eV)		
A	B	C
2.23	2.39	2.53

	PECD (%)		
*h*ν (eV)	A	B	C
3.49	–4.9 ± 2.0 (6.3 ± 1.3)^a^	1.9 ± 1.3 (−1.1 ± 0.3)^a^	3.8 ± 1.5 (−3.5 ± 0.8)^a^
2.76	–3.8 ± 0.1	0.0 ± 0.4	1.7 ± 0.7
2.53	1.0 ± 0.2	3.1 ± 0.6	
2.38	3.5 ± 0.1		

a *S* enantiomer measurement shown in parentheses.

Opposite signs of PECD for the two enantiomers are observed for all peaks in each spectrum, except for peak B in the 2.76 eV spectrum, where the magnitude of PECD is effectively zero. Upon comparison of PECD measured at a photon energy (*h*ν) of 3.49 eV for the two enantiomers, the magnitude of PECD measured for the *S* enantiomer falls within the confidence interval of the *R* enantiomer measurement. The individual, repeated PECD measurements of the *R* enantiomer, at each photon energy, can be found in Figures S3–S6.

The PECD observed for the 3.49 eV spectrum provides a clear example of the dynamic behavior of this chiroptical effect. Despite the photodetachment being constant in molecular conformation and electronic transition, the spectrum shows fluctuations in the PECD across the three bands, including a sign change going from band A to band B. These PECD differences across the spectrum can be attributed to the influence of the kinetic energy of the departing electron and/or the probed vibrational state. Similar observations have been made for many chiral molecules, including methyloxirane, which was one of the first observations of the vibrational dependence of the PECD effect.^23^ However, within this single spectrum, it is not possible to separate the individual influences.

The kinetic energy of the electron can greatly influence the scattering of the electron across the molecular potential and can even lead to a vanishing PECD effect at high eKEs. Significant changes in the measured PECD value with a decrease in eKE are often observed, as the electron becomes a more sensitive probe of the molecular geometry due to the longer interaction time of the photoelectron with the molecular potential. In Figure 4, the measured PECD effect for each band is shown in terms of eKE. Each spectral band does show a change in PECD with a change in eKE. Most obviously, band A shows a change in the sign of the PECD over a short energy interval, at low kinetic energies. To date, PECD in anions has only been experimentally studied at nominally low kinetic energies (<3 eV). However, nonvanishing PECD effects for anions have been predicted for higher eKEs.^32^ It would be interesting to utilize anions experimentally to investigate how quickly the chiral effect dissipates for increasing electron kinetic energies, free from the compensation of long-range interactions.

**Figure 4:**
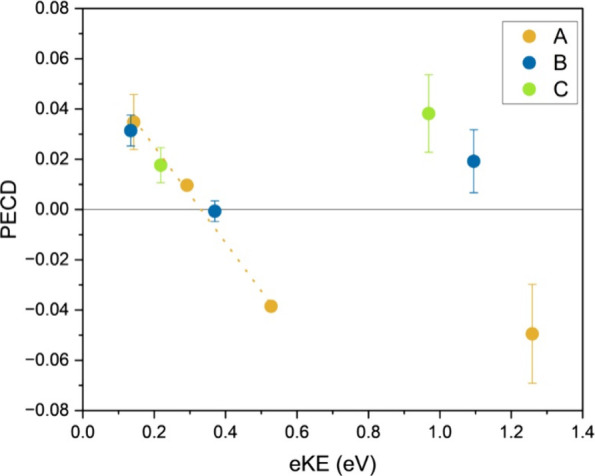
Measured PECD for peaks A (orange), B (blue), and C (green) as a function of electron kinetic energy (eKE). The orange dotted line marks the linear trend of the PECD for peak A in the eKE range from 0.14 to 0.53 eV.

Given the ubiquity of the ν_1_ mode throughout the photoelectron spectrum, it is not possible to analyze the explicit effect of this vibrational mode on the observed PECD. However, comparisons of band A with bands B and C can provide some insight into the potential influence the additional vibrational modes have on the PECD. In Figure 4, at higher eKEs, there are not enough data points to draw a clear conclusion about the relation of the PECD for the three spectral bands. However, it is worth noting that the PECD for the three bands at eKEs of >0.9 eV are all from the 3.49 eV photoelectron spectrum, where potential resonant states were observed. Interaction with resonant states has been previously shown to cause significant deviations in the measured PECD.^15,44,45^ At kinetic energies of <0.6 eV, peaks B (and C) follow the same seemingly linear trend in PECD as peak A. From these data, it is tempting to conclude that the additional vibrational content does not have an effect on the PECD.

Interestingly, our measurements of PECD for another chiral alcohol anion, deprotonated indanol, show a similar picture at low eKEs.^36^ For the O-deprotonated tautomer of indanol, three partially resolved vibrational contours are observed. At eKEs of ≤0.8 eV, the PECD trends of these three features are almost identical. The trend observed is similar to that of phenylethanol with a PECD sign change over a similar eKE range.^a^ Both anions have similar electronic properties, where photodetachment is occurring from a HOMO/HOMO–1 orbital that is oxygen-centered, which could explain the similarities in observed PECD.

Through photoelectron spectroscopy and complementary electronic structure calculations, we have determined the photodetachment of deprotonated 1-phenylethanol is primarily composed of a single electronic transition channel for the O-deprotonated tautomer. The vibrational landscape of the spectrum is dominated by the ν_1_ mode, present as an isolated vibrational band (band A) and incorporated in combination vibrational bands (bands B and C). Other vibrational excitations are primarily comprised of C–H deformations of the phenyl ring and C–C stretches. PECD was measured at multiple photon energies to evaluate the dependence of PECD on eKE and vibrational activity. A change in the PECD with eKE was observed for each respective peak, but given assumptions regarding the trend of PECD at eKEs of <0.6 eV, the additional vibrations do not appear to significantly alter the PECD measured. As the outgoing electron’s kinetic energy is minimized, the photoelectron should become more susceptible to the influence of electron correlation and resonances, enabling observation of vibrationally sensitive chiral effects. With advancements in anion photoelectron spectroscopy, such as cryogenic cooling of anions and infrared pumping, future experiments of this topic could provide well-controlled explorations of the short-range manifestation of the PECD effect.

## Experimental and Computational Methods

The anion photoelectron imaging spectrometer used in this work has been previously described.^36,46^ Deprotonated phenylethanol anions were produced via a plasma entrainment source, in which a pulsed expansion of enantiopure (*R*)- or (*S*)-1-phenylethanol seeded in Ar buffer gas is crossed with a pulsed plasma beam. OH^–^ present in the plasma beam abstracts a proton from the phenylethanol, producing a molecular beam containing deprotonated chiral anions.^47^ The vapor pressure of phenylethanol allows for incorporation into the buffer gas beam at room temperature, but heating the phenylethanol reservoir to ∼60 °C produced the largest amount of deprotonated phenylethanol. Mass-selected anions are photodetached using the third harmonic of a Nd:YAG laser (355 nm) or the output of a pulsed OPO (∼5 ns pulse duration, ∼0.4 mJ pulse energy) laser operating in the range of 2.38–2.75 eV. Linearly polarized light is utilized for the measurement of photoelectron spectra, and a shot-to-shot switching of the circularly polarized light is used in the measurement of PECD. Photodetached electrons are focused onto a position-sensitive detector, using velocity map imaging ion optics. The kinetic energies of imaged electrons are calibrated using the known electron detachment energies of the atomic sulfur anion (S^–^).

Anion photoelectron spectra are reconstructed using polar onion peeling.^48^ PECD is analyzed using a pBasex program created by Garcia et al., in which an Abel inversion of the difference image (i.e., LCP–RCP) is performed.^49^ LCP is defined by a counterclockwise rotation of the electric fields when facing the light source. The error in the PECD measurements of the anion PhEtO^–^ is calculated as the standard error of the mean (weighted by electron count) of all measurements taken at one photon energy with one enantiomer.

Electronic structure calculations of the isomers of the deprotonated anion of 1-phenylethanol were conducted by using density functional theory. The density functional theory (DFT) calculations utilized the hybrid density functional B3LYP^50−52^ with a Grimme’s D3 dispersion correction,^53^ and the augmented correlation-consistent polarized valence-only triple-ζ basis set (aug-cc-pVTZ).^54−56^ Optimized geometries and frequencies were obtained for the expected deprotonated tautomers and conformers of PhEtO^–^, as well as the corresponding dehydrogenated radical generated upon electron emission. The unscaled vibrational frequencies of the optimized geometries were used to conduct Franck–Condon simulations of the photoelectron spectra. The simulations assumed an experimental temperature of 100 K and a Gaussian line width of HWHM = 80 cm^–1^ (0.01 eV) for the individual transitions, to better match the resolution of the experimental spectra. For the main tautomer of PhEtO^–^, molecular orbitals and excited states of its resulting neutral radical were computed. Calculations of the excited states were conducted using time-dependent DFT, also with B3LYP-D3/aug-cc-pVTZ. A calculation of the natural transition orbitals was carried out to identify the state that modeled electron detachment from HOMO–1 of the anion. All calculations were carried out using Gaussian16.^57^

## Supporting Information Available

Molecular orbitals of the HOMO and HOMO–1 orbitals of deprotonated 1-phenylethanol, natural transition orbitals of the first excited state excitation of dehydrogenated 1-phenylethanol, starting structures for the geometry optimization of deprotonated 1-phenylethanol, tables of the Franck–Condon simulated transitions and vibrational frequencies of the anion and neutral radical, graphs showing the individual PECD measurements, and the raw PECD difference images taken at each photon energy (PDF)

## Footnotes

a The correction of a mistake in the data acquisition procedure leads to opposite sign assignment for phenylethanol measurements compared to past measurements in indanol.
