# Supplementary material for: Photoelectron Circular Dichroism in the Photodetachment of Deprotonated 1-Phenylethanol
Source: J Phys Chem Lett. 2025 Feb 3;16(6):1478–84. doi: 10.1021/acs.jpclett.4c03636 (PMC11831663; doi:10.1021/acs.jpclett.4c03636)
Supplement: Supplementary file 1 — jz4c03636_si_001.pdf [file jz4c03636_si_001.pdf]

Supporting Information for “Photoelectron Circular Dichroism in the Photodetachment of Deprotonated 1-Phenylethanol”

Molecular orbitals and natural transition orbitals of the deprotonated anion of (*R*)-1-phenylethanol.

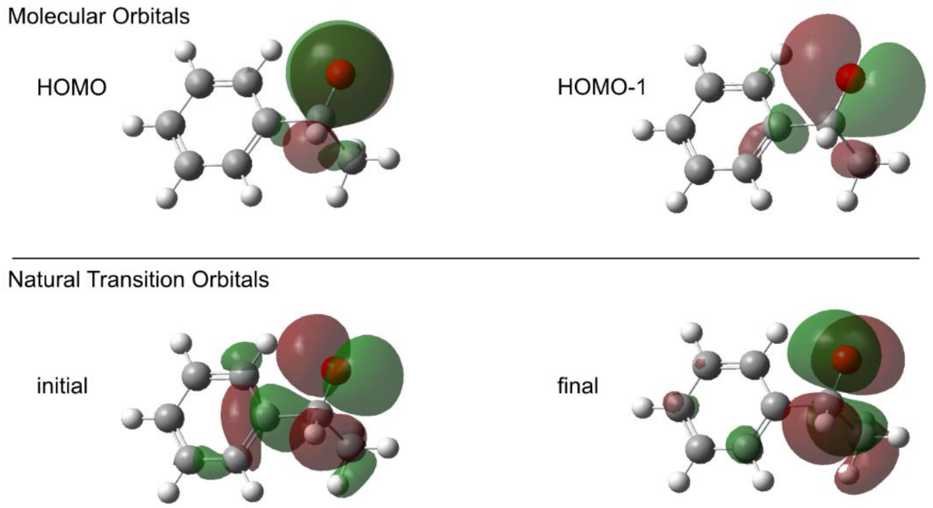

Figure S1: (top) Molecular orbitals: HOMO and HOMO-1 of the O-deprotonated anion of (R)-1-phenylethanol. (bottom) Natural transition orbitals calculated for the first excited state of the neutral radical formed in the photodetachment. The “initial” orbital defines the oribital where the electron is located, and the “final” orbital is the orbital where the electron is promoted. After electron promotion, a hole is created in the initial orbital. As the initial NTO ressembles the HOMO-1 orbital of the anion, it can be concluded that the first excited state of the neutral radical reflects electron detachment from the HOMO-1 orbital of the anion. Theses calculations were conducted with DFT/B3LYP-D3/aug-cc-pVTZ.

Geometry optimization of 1-PhEtO$^-$

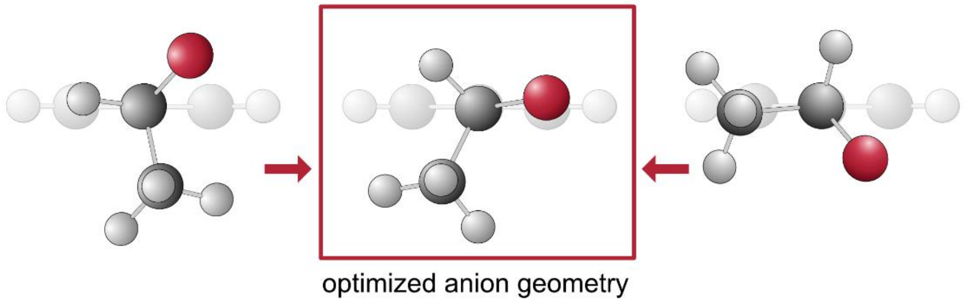

Figure S2: Initial geometries of the three expected conformations of PhEtO$^-$, where the CH bond, CO$^-$ bond, and C-methyl bond have been placed in the plane of the ring. All starting geometries optimize to the geometry where the CO$^-$ bond is nearly planar with the ring.

Table S1: Molecular structure of deprotonated anion at optimized geometry, given in cartesian coordinates.

	Coordinates (Angstrom)		
Atomic number	X	Y	Z
6	-0.27872	0.021685	-0.04009
6	-0.14069	0.008918	1.50669
6	1.070459	-0.22014	2.164269
6	1.152362	-0.22112	3.55328
6	0.010938	0.012004	4.319787
6	-1.20092	0.248974	3.675392
6	-1.27163	0.250883	2.284698
1	-2.18791	0.45017	1.741781
1	-2.0928	0.439792	4.263151
1	0.069494	0.015852	5.402036
1	2.104499	-0.39965	4.041625
1	1.965285	-0.39232	1.574987
6	0.056916	-1.42166	-0.55367
1	0.022464	-1.4011	-1.64451
1	1.038741	-1.79194	-0.23186
1	-0.71784	-2.1071	-0.19944
8	-1.45344	0.459165	-0.48516
1	0.605568	0.646974	-0.37158

Table of vibrational modes

Table S2: Vibrational modes that contribute most to band A, B and C, given with initial and final state, as well as, the relative intensity of the different quanta. The most intense quanta are highlighted in bold font.

Shifted Electron Detachment Energy (eV)	Initial State	Final State	Intensity
Peak A			
2.22993	|0>	|1^1>	952.8
2.23485	|0>	|1^2>	2038
2.23978	|0>	|1^3>	**2378**
2.2447	|0>	|1^4>	1578
2.24963	|0>	|1^5>	538
Peak B			
2.36454	|0>	|24^1;1^1>	119.2
2.36947	|0>	|24^1;1^2>	304.8
2.37439	|0>	|24^1;1^3>	**433.8**
2.37932	|0>	|24^1;1^4>	365.3
2.38424	|0>	|24^1;1^5>	173.8

<table>
<tr><td></td><td></td><td></td><td></td></tr>
<tr><td>2.3771</td><td>|0></td><td>|25^1;1^3></td><td>**94.67**</td></tr>
<tr><td>2.38203</td><td>|0></td><td>|25^1;1^4></td><td>85.09</td></tr>
<tr><td></td><td></td><td></td><td></td></tr>
<tr><td>2.38843</td><td>|0></td><td>|27^1;1^3></td><td>106.9</td></tr>
<tr><td>2.39336</td><td>|0></td><td>|27^1;1^4></td><td>**107.9**</td></tr>
<tr><td></td><td></td><td></td><td></td></tr>
<tr><td>2.3898</td><td>|0></td><td>|28^1;1^3></td><td>**102.7**</td></tr>
<tr><td>2.39473</td><td>|0></td><td>|28^1;1^4></td><td>84.57</td></tr>
<tr><td></td><td></td><td></td><td></td></tr>
<tr><td>2.39033</td><td>|0></td><td>|30^1;1^1></td><td>268.4</td></tr>
<tr><td>2.39525</td><td>|0></td><td>|30^1;1^2></td><td>584.5</td></tr>
<tr><td>2.40018</td><td>|0></td><td>|30^1;1^3></td><td>**697**</td></tr>
<tr><td>2.40511</td><td>|0></td><td>|30^1;1^4></td><td>476</td></tr>
<tr><td>2.41003</td><td>|0></td><td>|30^1;1^5></td><td>170</td></tr>
<tr><td>2.41628</td><td>|0></td><td>|30^1;2^1;1^3></td><td>79.99</td></tr>
<tr><td></td><td></td><td></td><td></td></tr>
<tr><td>2.40226</td><td>|0></td><td>|31^1;1^2></td><td>152.9</td></tr>
<tr><td>2.40718</td><td>|0></td><td>|31^1;1^3></td><td>**182.6**</td></tr>
<tr><td>2.41211</td><td>|0></td><td>|31^1;1^4></td><td>124.9</td></tr>
<tr><td></td><td></td><td></td><td></td></tr>
<tr><td>2.40154</td><td>|0></td><td>|33^1;1^1></td><td>192.3</td></tr>
<tr><td>2.40646</td><td>|0></td><td>|33^1;1^2></td><td>411.7</td></tr>
<tr><td>2.41139</td><td>|0></td><td>|33^1;1^3></td><td>**480.8**</td></tr>
<tr><td>2.41631</td><td>|0></td><td>|33^1;1^4></td><td>319.4</td></tr>
<tr><td>2.42124</td><td>|0></td><td>|33^1;1^5></td><td>109.2</td></tr>
<tr><td colspan="4">Peak C</td></tr>
<tr><td>2.52987</td><td>|0></td><td>|30^1;24^1;1^2></td><td>88.95</td></tr>
<tr><td>2.5348</td><td>|0></td><td>|30^1;24^1;1^3></td><td>**128.7**</td></tr>
<tr><td>2.53972</td><td>|0></td><td>|30^1;24^1;1^4></td><td>110.8</td></tr>
<tr><td>2.546</td><td>|0></td><td>|33^1;24^1;1^3></td><td>92.25</td></tr>
<tr><td></td><td></td><td></td><td></td></tr>
<tr><td>2.55566</td><td>|0></td><td>|30^2;1^2></td><td>88.79</td></tr>
<tr><td>2.56058</td><td>|0></td><td>|30^2;1^3></td><td>**108**</td></tr>
<tr><td></td><td></td><td></td><td></td></tr>
<tr><td>2.56686</td><td>|0></td><td>|33^1;30^1;1^2></td><td>122</td></tr>
<tr><td>2.57179</td><td>|0></td><td>|33^1;30^1;1^3></td><td>**145.6**</td></tr>
<tr><td>2.57672</td><td>|0></td><td>|33^1;30^1;1^4></td><td>99.52</td></tr>
</table>

Vibrational modes of the deprotonated anion 1-PhEtO$^-$ and the corresponding neutral radical formed by photodetachment

Table S3: Vibrational modes of 1-PhEtO$^-$ and associated IR intensities.

	Frequency (cm^{-1})	IR intensity
1	64.3425	4.5885
2	134.3282	0.7909
3	216.8703	9.7393
4	256.927	1.1573
5	318.8796	5.485
6	385.9017	6.259
7	420.6748	0.2294
8	468.4599	4.7953
9	529.9865	7.7356
10	629.8585	8.8961
11	633.0183	0.4083
12	716.698	41.6999
13	733.1814	20.7242
14	766.3817	17.9424
15	843.5138	14.4763
16	859.1985	0.4288
17	917.6422	3.1304
18	981.2879	1.1366
19	1001.216	3.0076
20	1013.301	8.8661
21	1022.883	9.7773
22	1043.399	14.2667
23	1052.414	50.6116
24	1074.437	17.5619
25	1142.35	1.541
26	1170.641	1.793
27	1186.037	2.7976
28	1209.155	182.7424
29	1279.22	51.9064
30	1301.725	24.9443
31	1333.317	8.7061
32	1341.617	4.9107
33	1349.894	36.6024
34	1459.875	1.5526
35	1468.701	8.5883
36	1480.691	1.9734

37	1506.161	15.0618
38	1607.224	6.2781
39	1618.069	22.4333
40	2545.663	387.4146
41	2962.874	97.6649
42	3036.791	63.6848
43	3067.766	60.8933
44	3112.421	9.2944
45	3121.557	14.7677
46	3133.663	78.6238
47	3151.528	68.0337
48	3160.784	31.0203

Table S4: Vibrational modes of the neutral radical, formed by deprotonation of 1-PhEtO$^-$, and associated IR intensities.

	Frequency (cm^{-1})	IR intensity
1	39.7281	2.0316
2	129.8217	1.306
3	208.7879	1.7648
4	253.7373	1.0779
5	296.8532	0.1157
6	359.0973	4.3274
7	414.3683	0.1356
8	444.8378	2.0924
9	542.9932	14.4691
10	605.067	4.2651
11	634.1965	0.0106
12	719.1886	40.1684
13	764.6747	2.3158
14	777.7016	24.0878
15	864.9135	2.2799
16	870.2992	8.7283
17	926.1543	2.4015
18	942.5842	3.492
19	998.9313	0.1276
20	1016.916	0.139
21	1020.379	0.1358
22	1043.365	5.7009
23	1050.294	5.1806
24	1085.754	8.2164

25	1107.593	6.2909
26	1189.775	0.1011
27	1198.974	4.0402
28	1210.059	0.3519
29	1239.201	14.4202
30	1293.737	11.9008
31	1350.219	1.6151
32	1373.066	1.1056
33	1384.134	7.6158
34	1471.474	3.6638
35	1487.841	14.5293
36	1495.682	0.5907
37	1529.306	7.9006
38	1626.588	0.6115
39	1641.953	4.3993
40	2920.318	20.7764
41	3045.574	11.7577
42	3128.687	9.803
43	3136.468	16.1861
44	3146.119	8.0116
45	3162.581	0.1646
46	3172.966	14.2841
47	3184.085	15.2157
48	3190.896	11.5164

Reproducibility across different measurements for all wavelengths

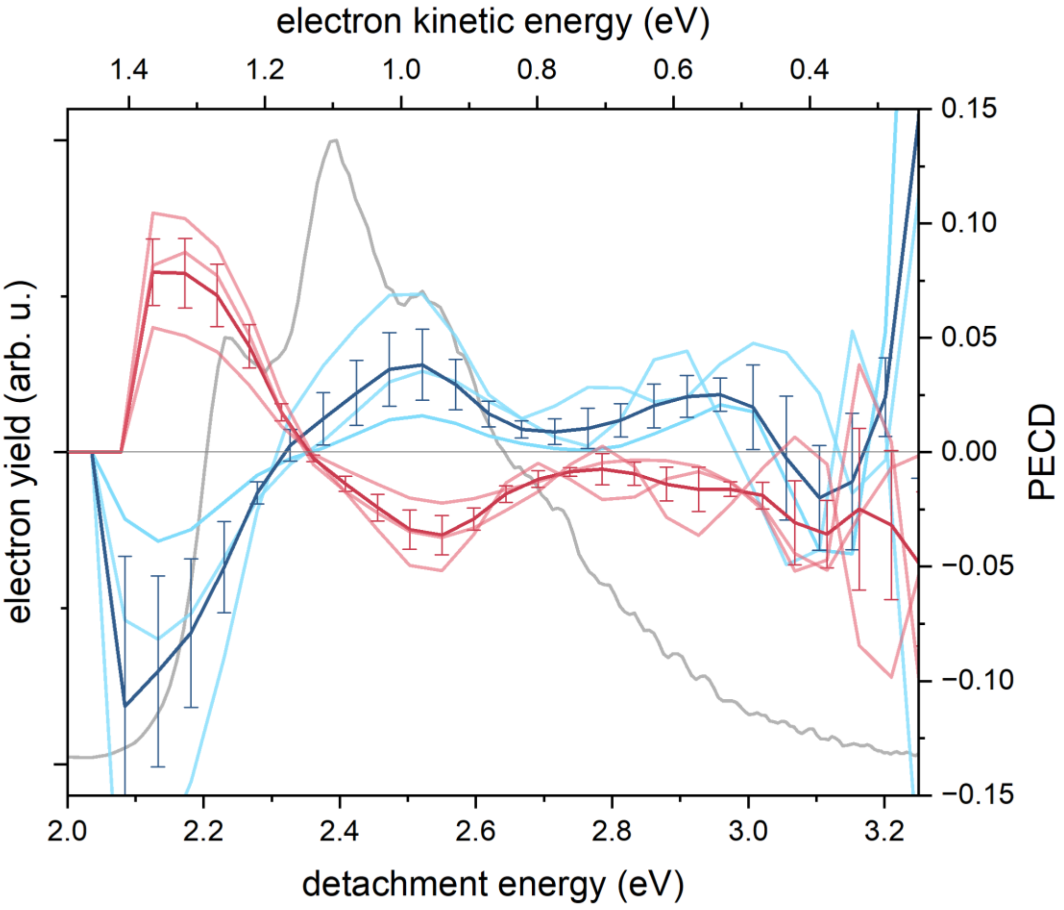

Figure S3: Photoelectron spectrum (grey) and PECD (blue: (R) and red: (S)) of $PhEtO^-$ anion measured at hν = 3.49 eV. Single measurements are shown in light blue and red, respectively. The weighted average of each set of measurements, including the standard error, are shown in darker blue and red.

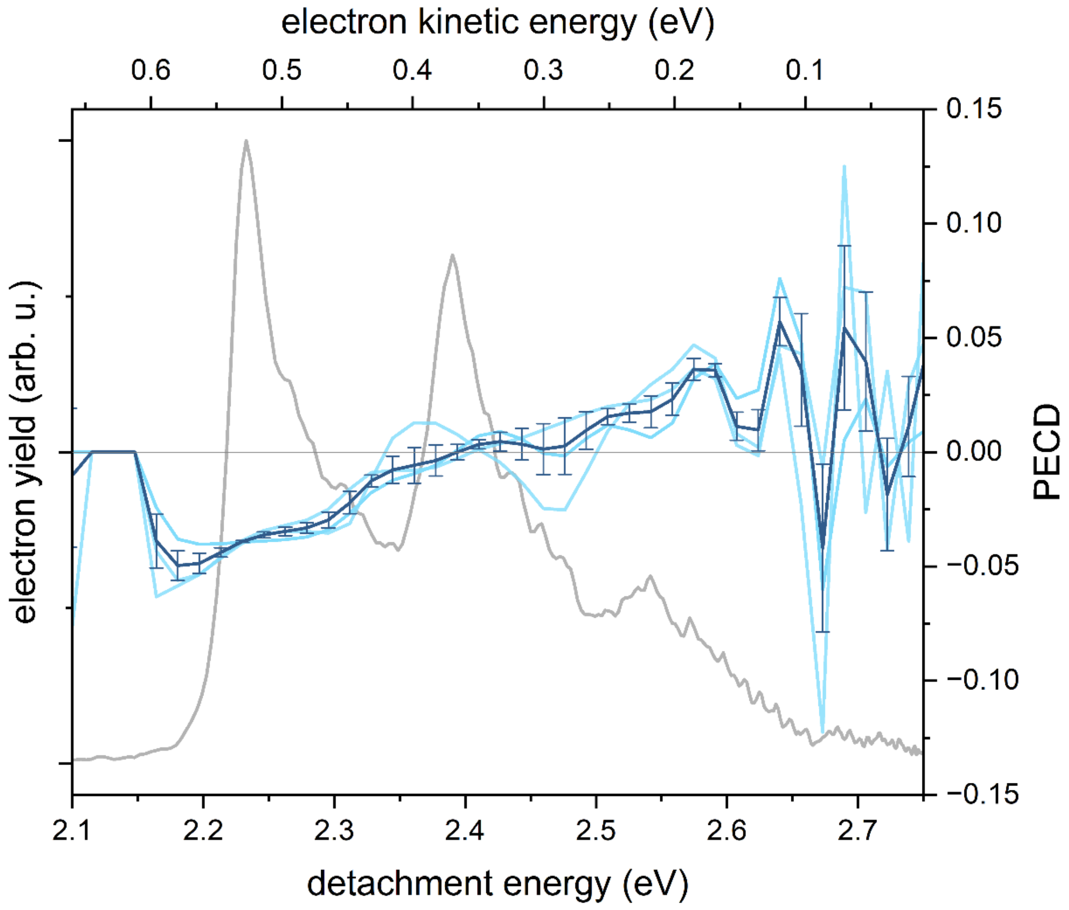

Figure S4: Photoelectron spectrum (grey) and PECD (blue) of (R)-PhEtO$^-$ anion measured at hν = 2.76 eV. Single measurements are shown in light blue. The weighted average including the standard error are shown in dark blue

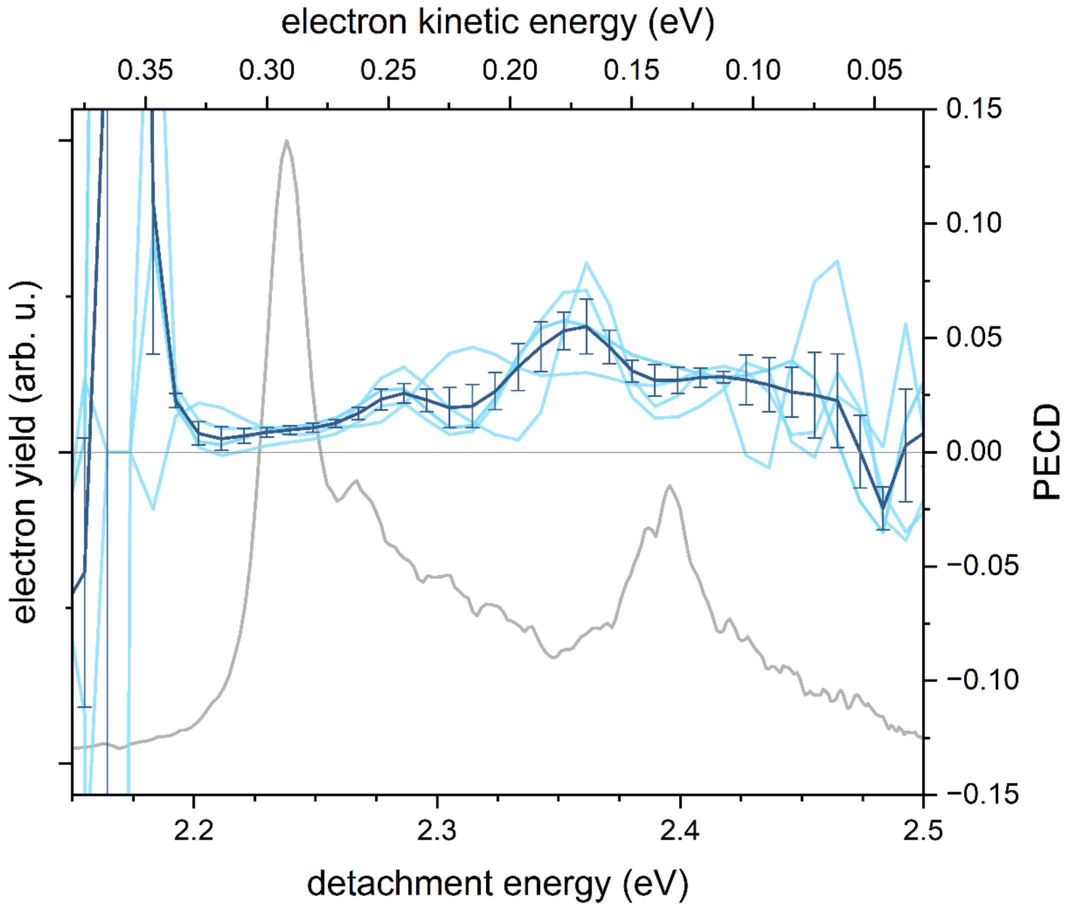

Figure S5: Photoelectron spectrum (grey) and PECD (blue) of (R)-PhEtO⁻ anion measured at hν = 2.53 eV. Single measurements are shown in light blue. The weighted average including the standard error are shown in dark blue

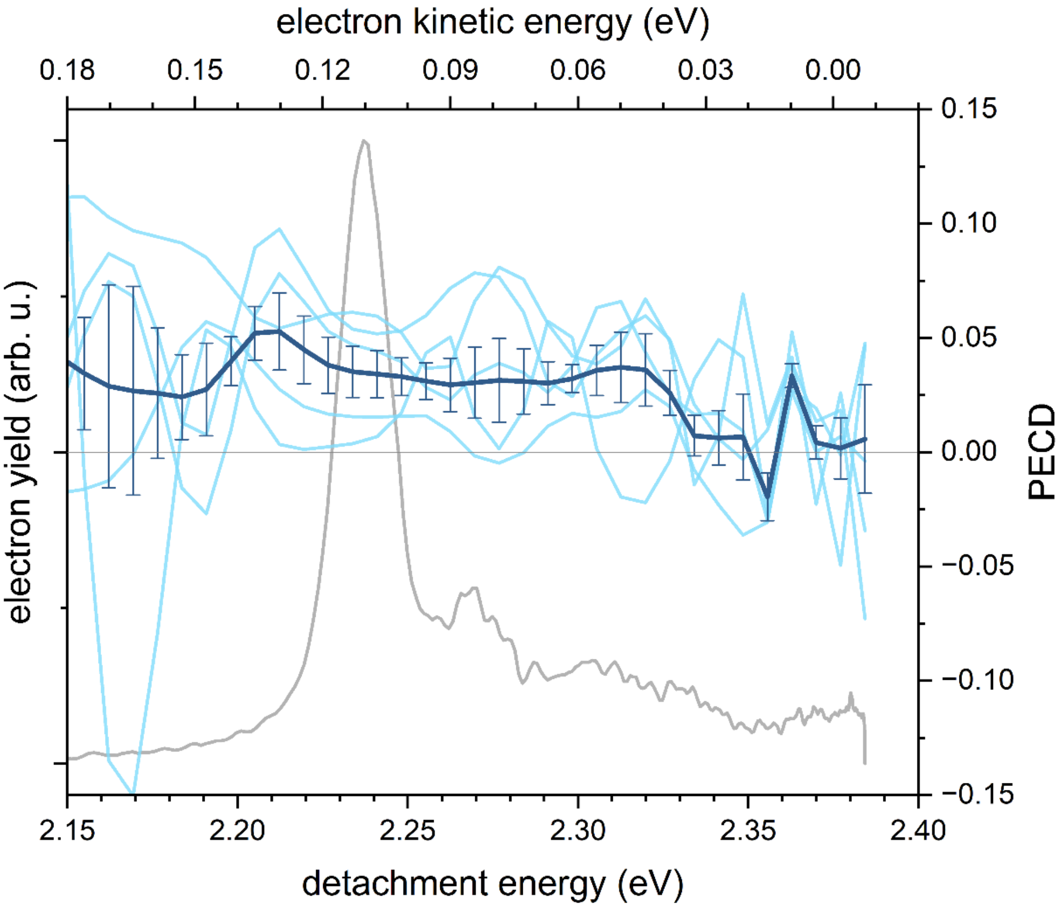

Figure S6: Photoelectron spectrum (grey) and PECD (blue) of (R)-PhEtO$^-$ anion measured at hν = 2.38 eV. Single measurements are shown in light blue. The weighted average including the standard error are shown in dark blue.

Photoelectron difference images of 1-PhEtO$^-$

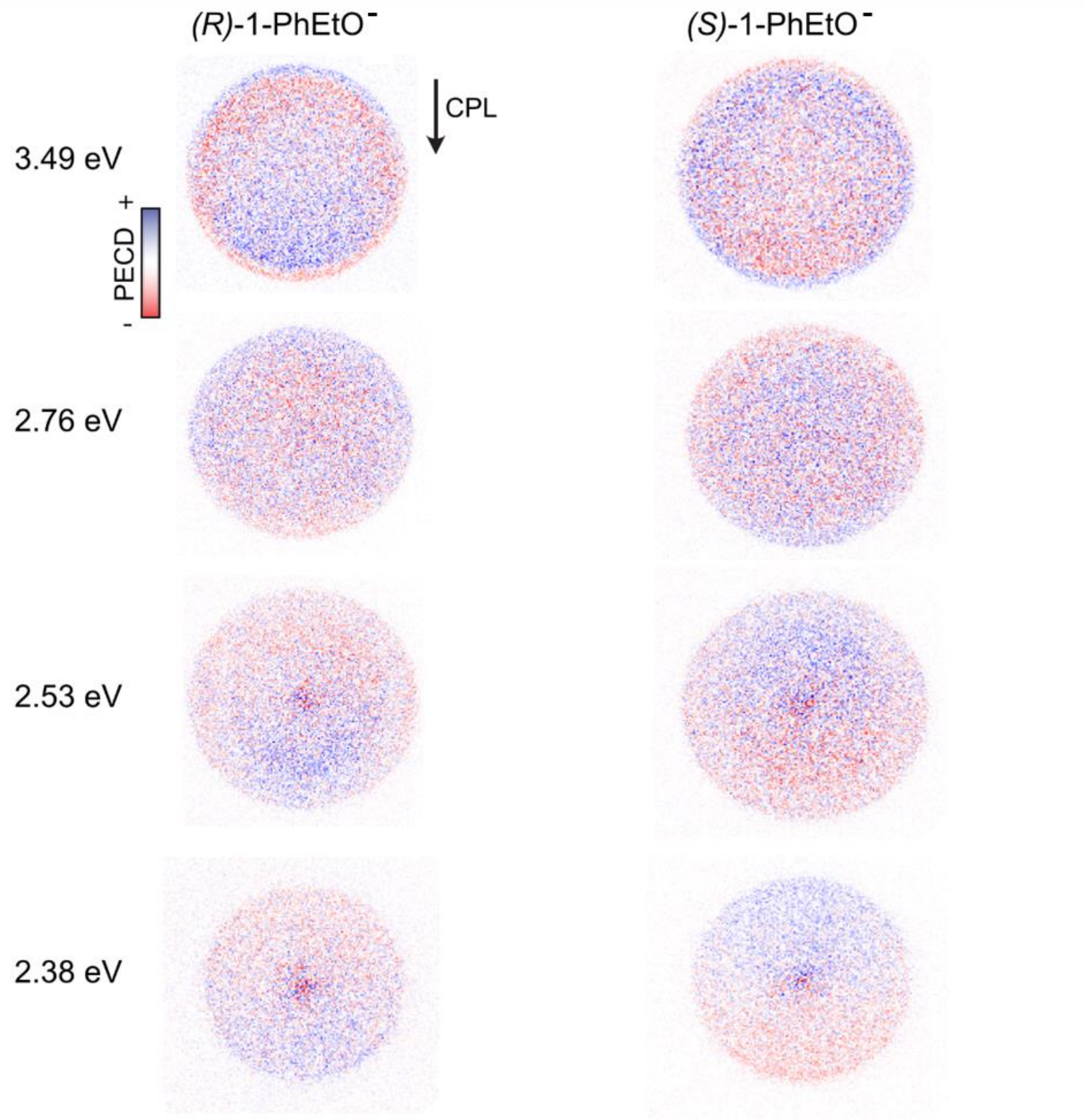

Figure S7. Raw difference (LCP-RCP) images of the photoelectron angular distributions for both enantiomers of dehydrogenated 1-phenylethanol, at the four measured photon energies (i.e. 3.49, 2.76, 2.53, and 2.38 eV).
